# Harnessing Plant-Derived Terpenoids for Novel Approaches in Combating Bacterial and Parasite Infections in Veterinary and Agricultural Settings

**DOI:** 10.1007/s00284-025-04113-4

**Published:** 2025-02-12

**Authors:** Danielle Wiles, Jaclyn S. Pearson, Travis Beddoe

**Affiliations:** 1 Department of Animal, Plant and Soil Sciences and AgriBio Centre for AgriBioscience, School of Agriculture, Biomedicine and Environment, La Trobe University, Bundoora, VIC 3083 Australia; 2 Australian Research Council Research Hub for Medicinal Agriculture, AgriBio Centre for AgriBioscience, La Trobe University, Bundoora, VIC 3086 Australia; 3 Centre for Innate Immunity and Infectious Diseases, Hudson Institute of Medical Research, Clayton, VIC 3168 Australia; 4 Department of Microbiology, Monash University, Clayton, VIC 3168 Australia; 5 School of Medicine, University of St Andrews, St Andrews, Fife, KY16 9TF UK

## Abstract

The rising resistance to conventional antimicrobial therapies in veterinary contexts poses a formidable challenge. While various innovative treatment strategies for pathogenic infections have emerged, their success still needs to be improved, warranting continued research. Recent investigations into natural products as potential sources for biologically active therapeutics have gained traction. Phytochemicals present a promising alternative in combating a spectrum of pathogens, including bacteria, fungi and parasites. One such class of phytochemicals with mounting potential is the structurally diverse terpenes. These chemicals contribute to plants’ characteristic odour and medicinal effects and have been widely investigated in the scientific literature for their exceptional antibacterial activity. Their efficacy is demonstrated through diverse mechanisms, encompassing damage to bacterial membranes, suppression of virulence factors, and interference with enzymes, toxins, and biofilm formation. This review comprehensively examines terpenes’ in vitro and in vivo activity and their derivatives against pathogens, elucidating their potential against antimicrobial resistance (AMR) and the underlying mechanisms specific to each terpene class. The findings underscore the burgeoning potential of terpene therapy as a viable alternative or supplementary approach to conventional antibiotics in addressing bacterial and parasitic infections in livestock and companion animals.

**Supplementary Information:**

The online version contains supplementary material available at 10.1007/s00284-025-04113-4.

## Introduction

### Antimicrobial Resistance

The veterinary industry faces significant challenges in addressing bacterial and parasitic infections among animals, posing threats to animal health, welfare, and the economy. Since their discovery in 1928, traditional antimicrobial and anthelmintic therapies have faced a substantial challenge with the emergence of antimicrobial resistance (AMR) [1]. AMR, characterised by diminished effectiveness of antimicrobial agents due to prolonged antibiotic exposure, is fuelled by intrinsic microbial features, genetic mutations, and extrinsic factors, including excessive antibiotic use in humans and animals and inadequate sanitation and education [2].

AMR in the veterinary industry has been a subject of increasing concern, with Laxminarayan et al. [3] reporting that antibiotic usage in growth and disease prevention in the veterinary, agriculture, aquaculture, and horticulture industries are the main contributors in the non-medical clinical setting. Animals, spanning production, companion, and exotic species share ecosystems with humans, thus heightening the risk of disease transmission [2]. While antibiotics have greatly improved animal health and production effectiveness, their misuse and overuse in veterinary medicine pose significant AMR challenges [3]. In addition, companion animals, particularly dogs and cats, have emerged as potential AMR reservoirs in recent years, emphasising the need for judicious antibiotic use, responsible diagnosis, and global collaborative efforts in developing new antimicrobial agents [3]. Addressing AMR in the veterinary sector is vital for animal health and welfare and imperative for safeguarding human health, which is intricately linked to the well-being of animals.

### Plant-Derived Natural Products

The demand for innovative pathogen treatments is unmistakable, and there has been a significant upsurge in exploring natural antibiotic alternatives, including probiotics, organic acids, prebiotics, enzymes, bacteriophages, and phytochemicals [4]. However, despite acknowledging their benefits, a more comprehensive understanding of the mechanisms of action, efficiency, and practical application of these therapeutic alternatives is still needed [5].

Phytochemicals have emerged as a particularly promising avenue in the search for alternative antibiotic sources [5]. These compounds exhibit antimicrobial and antioxidant properties and even exhibit actions stabilising microbiota and facilitating immune modulation [6]. As the quest for innovative pathogen treatments in animal agriculture and companion animals intensifies, phytochemicals stand out as promising antibiotic alternatives.

### Terpenes and Their Derivatives

**Fig. 1:**
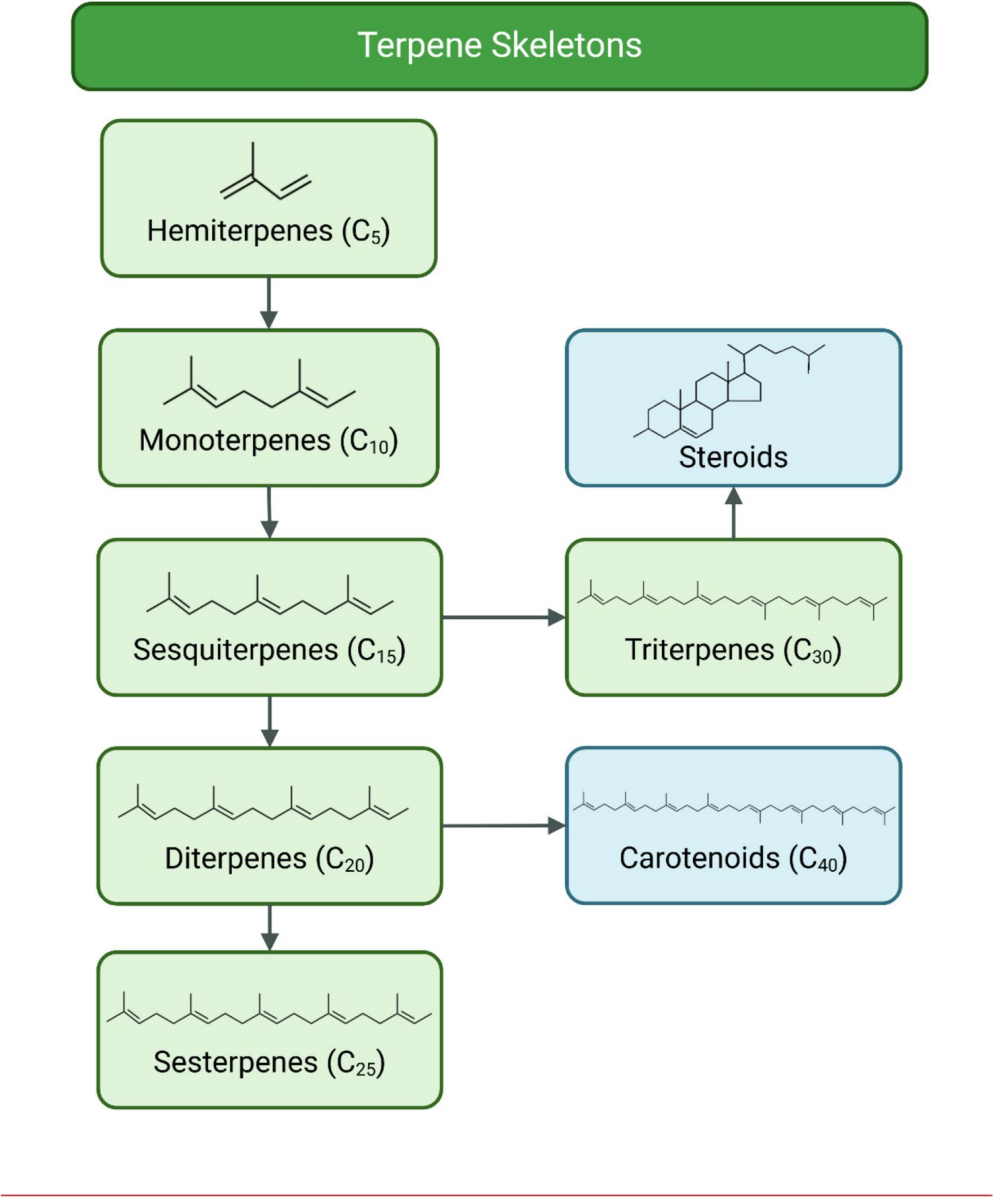
*Structural diversity* of *core carbon skeletons defining* terpene *classes*

Terpenes and their terpenoid derivatives are a class of natural compounds found in a wide range of plant species, constituting the largest class of natural products with > 80,000 known structurally diverse compounds, as listed in the Dictionary of Natural Products (http://dnp.chemnetbase.com). Derived from the isoprenoid biosynthesis pathway, terpenes are the main components of essential oils and exist in various forms based on the number of carbons in their backbone (hemiterpenes (C_5_), monoterpenes (C_10_), sesquiterpenes (C_15_), diterpenes (C_20_), sesterterpenes (C_25_), triterpenes (C_30_), and tetraterpenes (C_40_), influencing their chemical functionalities (Fig. 1) [7]. While terpenes are simple hydrocarbons, terpenoids have undergone natural biochemical modifications via enzymes, wherein oxidised methyl groups are removed or replaced (Supplementary Figure S1). However, terpenoids can be further subdivided into alcohols, aldehydes, ethers, esters, ketones, phenols, and epoxides.

Many terpenes are extensively applied commercially as flavours, fragrances, cosmetic products, and food additives. In the pharmaceutical industry, there is a growing interest in the clinical application of terpenoids, both as excipients to enhance skin or membrane penetration or as active principles of drugs [8]. A broad range of biological properties have been described for terpenes, including cancer chemo-preventative effects, antifungal, antiviral, antihyperglycemic, analgesic, anti-inflammatory, antimicrobial, and antiparasitic activities [9]. Terpenoids have historically been used in traditional medicine and have recently inspired terpene-based pharmaceutical development. Among these, the anticancer (Paclitaxel; [10]) and antimalarial (Artemisinin; [11]) drugs are two of the most renowned terpene-based drugs, highlighting the potential of terpenoids as therapeutic agents. Overall, terpenes are well suited for industrial applications due to their availability and synthesis, representing a promising avenue for developing novel antimicrobial and antiparasitic therapies.

## Terpenes as Antimicrobial Agents

Terpenes hold significant promise as antimicrobial alternatives in veterinary medicine due to their diverse bactericidal properties [12]. They demonstrate a broad spectrum of activity against bacteria, disrupting microbial cell membranes, affecting membrane permeability, and interfering with essential cellular processes [12]. This is particularly important in veterinary medicine, where AMR in animals can impact both animal and public health.

**Fig. 2:**
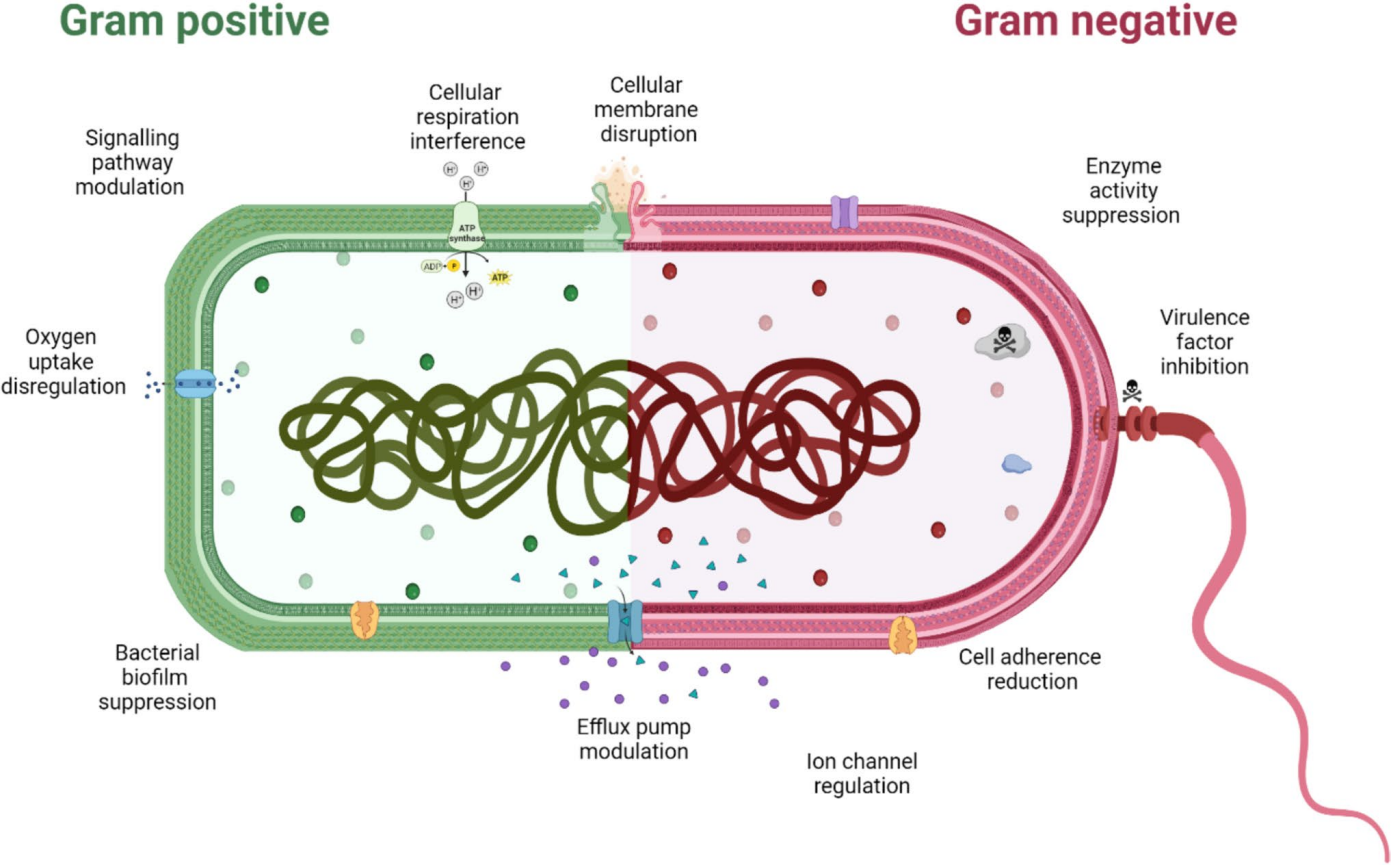
Terpene antimicrobial mechanisms of action on Gram-positive and Gram-negative bacteria

In general, terpenes exhibit differential effects on Gram-positive and Gram-negative bacteria, with Gram-positive bacteria having greater antimicrobial sensitivity due to differences in membrane structure, permeability, composition, and charge (Fig. 2). However, there are reports of terpenes displaying potent antibacterial effects against Gram-positive and Gram-negative bacteria, offering potential solutions to AMR.

### Terpene Activity in Gram-Positive Bacterial Infections

**Fig. 3:**
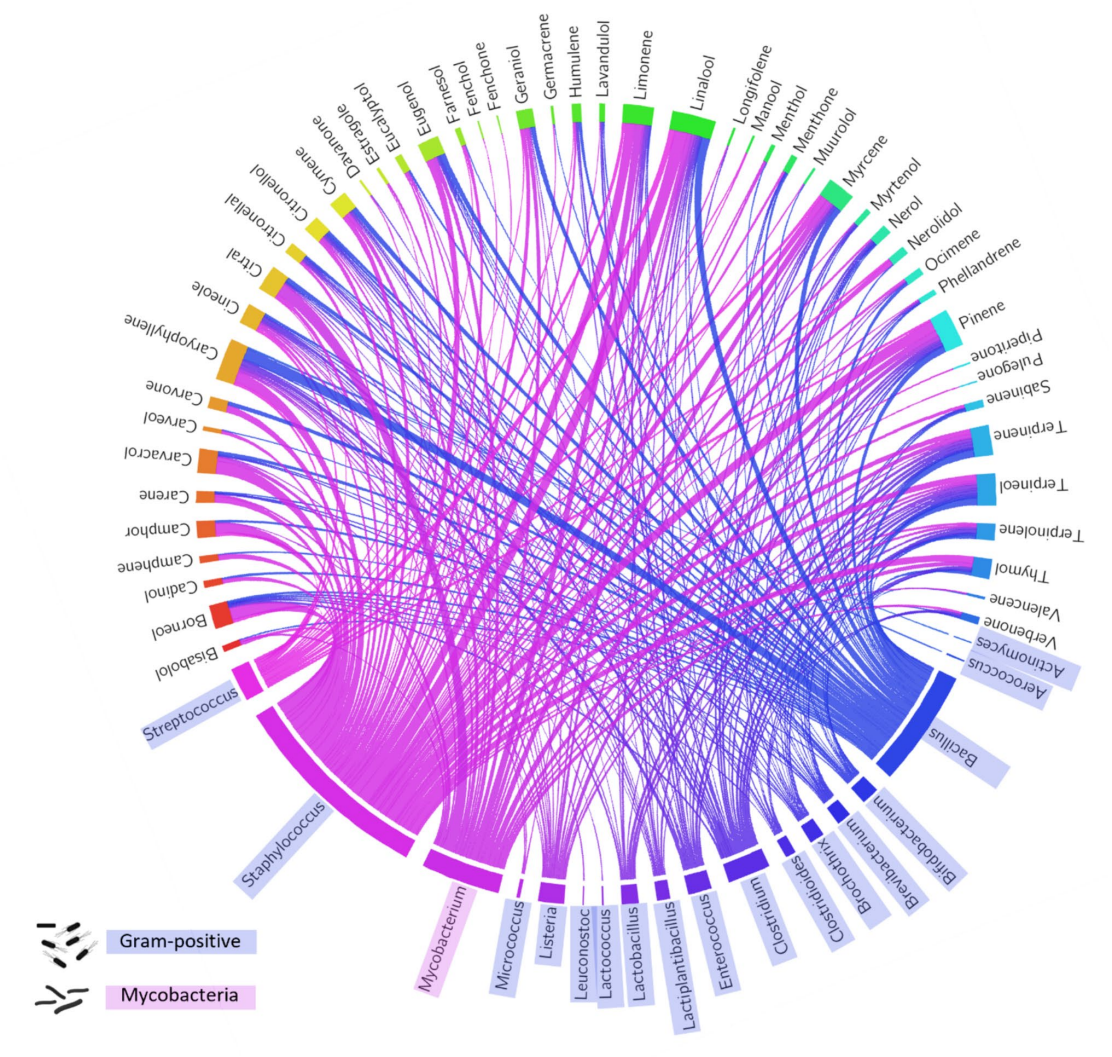
Interaction between the diversity of terpenoids and Gram-positive bacteria based on a survey of peer-reviewed articles on antimicrobial terpenes. The circus plot illustrates the relationship between various terpenoids tested for their antibacterial activity against specific Gram-positive bacteria (including mycobacterial species), as reported in the literature. The data were compiled from a systematic survey of studies examining the antimicrobial activity of terpenoids, and the connections in the plot highlight the terpenes tested against each bacterial genus. The plot was generated using the Circos visualisation software, with the input dataset derived from the data available in the supplementary materials

Gram-positive infections are a significant concern in the veterinary industry, impacting various animals and posing considerable health and economic concerns [13]. Amid growing AMR resistance, terpenoids have demonstrated antimicrobial activity against various gram-positive pathogens (Fig. 3). Their antimicrobial efficacy against Gram-positive pathogens is rooted in their ability to target the thicker peptidoglycan layer characteristic of Gram-positive bacterial cell walls, which provides not only structural integrity but also virulence (Fig. 2) [14]. This vulnerability makes Gram-positive bacteria more susceptible to terpenes’ actions. Terpenes also exhibit other diverse mechanisms of action, demonstrating promise in disrupting capsules or biofilms, common defences of Gram-positive bacteria, which often pose challenges to conventional antibiotics [14]. Their capability to counteract hydrolase enzymes like β-lactamases, involved in antibiotic resistance, suggests terpenes may offer solutions against resistant strains [14].

The use of terpenes in treating Gram-positive bacterial infections has undergone extensive examination (Supplementary 1). While earlier literature predominantly explored plant extracts, recent emphasis has shifted towards investigating individual terpene components. From this work, several terpenes and their derivatives have emerged as promising candidates, including terpinolene [15], limonene [16], β-caryophyllene [17], linalool [18], α-pinene [19], nerolidol [20], camphor [21], thymol [12], carvacrol [12], and menthol [22] exhibiting antibacterial and antibiofilm activities against various Gram-positive strains.

Contemporary research has again refocused on unravelling the structural determinants and mechanistic insights contributing to terpenoids’ antimicrobial activity, focussing on how the functional groups of terpenoids contribute to bacterial death [12]. Key recurring themes encompass the role of phenolic structures, stereochemistry, and oxygenated terpenes in augmenting antimicrobial activity. Within the literature, phenolic compounds such as carvacrol, thymol, and eugenol consistently exhibit robust bactericidal effects against a broad spectrum of Gram-positive bacteria [12]. The hydroxyl group in phenolic structures significantly influences antimicrobial activity and is thought to form hydrogen bonds with target enzymes, leading to membrane dysfunction and bacterial death [23]. The addition of alkyl groups to phenolic compounds can also increase their hydrophobicity, making them less attracted to water and more prone to interacting with bacterial cells. This increased hydrophobicity reduces the surface tension of bacterial cells, facilitating better penetration of the phenolic compounds [24]. Previously, the action of terpenoids has primarily been attributed to the lipophilicity of terpenoids, hypothesising that terpenoids with low water solubility will show limited activity [14]. However, carvacrol and thymol are unusual in that they are strongly active despite their relatively low capacity to dissolve in water [12]. Beyond phenols, other terpenoid classes also exhibit antibacterial potential, including aldehydes, ketones, and alcohols. The electronegativity of aldehydes, particularly those conjugated to carbon-to-carbon double bonds, is proposed to contribute to their potent antimicrobial effects [24], suggesting an increase in electronegativity may interfere with biological processes involving electron transfer and react with vital nitrogen components, e.g. proteins and nucleic acids and therefore inhibit the growth of the microorganisms [14]. In the case of ketones, the presence of an oxygen function in the framework increases the antimicrobial properties of terpenoids [25]. Alcohol terpenoids potentially act as either protein-denaturing agents, solvents or dehydrating agents of microbes. The biological activity of menthol has been comprehensively reviewed by Kamatou et al., [26] highlighting its recognition as an effective antimicrobial agent against a diverse range of microorganisms. As a cyclic monoterpene alcohol, menthol exhibits its antimicrobial activity similarly to other terpenoids, primarily through its ability to disrupt bacterial cell membranes. This mode of action involves increasing membrane permeability, which leads to the leakage of intracellular contents and ultimately results in cell death. The mechanism is closely linked to menthol’s hydrophobic nature, which allows it to integrate into lipid bilayers and compromise their structural integrity. Additional factors are at play, such as the introduction of acetate moieties, carbonylation, and stereo-selectivity being linked to increased activity, with α-isomers being less active than β-isomers [27].

Notably, terpenes exhibit promise in addressing antibiotic-resistant bacterial strains, particularly against the pervasive threat of *Staphylococcus aureus.* The veterinary sector, grappling with the challenge of staphylococcal infections, including Methicillin-Resistant *S. aureus* (MRSA), has seen favourable results. Kuzma et al. [28] and Gupta et al. [29] showcased sesquiterpenes and diterpenes, such as clerodane diterpenoid (CD) and salvipisone, as potent enhancers of antibiotic efficacy in vitro against MRSA. Both compounds exhibited significant antibacterial and antibiofilm activities, reducing the minimum inhibitory concentrations (MICs) of antibiotics used in combination therapies. Additionally, triterpenoids, like amyrin, betulinic acid, and betulinaldehyde, also displayed potent antimicrobial activities against MRSA and Methicillin-Susceptible (MSSA) strains, making them potential therapeutic candidates [30]. Further research by Zdravkovic et al. [31] highlighted the strong antimicrobial effects of terpenes like carvacrol against MRSA, suggesting their viability as alternatives to combat AMR. There are also additional studies on terpenoids, with compounds like nerolidol, limonene, and β-caryophyllene consistently emerging as reliable inhibitors of this significant pathogenic threat to the veterinary industry [32].

The veterinary industry encounters a substantial burden of bacterial skin infections in companion animals, primarily stemming from the normal resident microflora. Studies by Budgin et al. [33] and Nocera et al. [34] highlight the efficacy of terpenes like linalool and limonene against *Staphylococcus pseudintermedius*, a multi-drug resistant pathogen implicated in skin infections in dogs and cats. Farnesol, a natural sesquiterpenoid, exhibits notable antibacterial effects against *Staphylococcus epidermidis*, suggesting therapeutic potential [35]. Similarly, combining farnesol and xylitol further inhibited *S. aureus* biofilm production without disrupting normal skin flora [36]. These findings bear significance for veterinary dermatology, particularly in light of rising antibiotic resistance. In addition to dermatological infections, wound infections pose a significant concern in veterinary medicine. Traditional treatments involve topical wound care and antibiotics, but interest in alternative therapies, including terpenes, is growing. When topically applied, terpenes and their derivatives offer potential in treating veterinary wound infections due to their natural antimicrobial properties and ability to modulate wound healing processes [37]. Compounds like α-pinene and β-pinene exhibit antimicrobial prowess against Gram-positive bacteria, making them potential candidates for disinfectants and wound infection treatments [12].

Beyond targeting specific pathogens, terpenoids offer a versatile solution for treating gastrointestinal (GI), respiratory, and cardiovascular infections. Research on the ability of terpenes to inhibit commonly isolated GI bacterial pathogens suggests their potential application in veterinary medicine [38]. In respiratory infections, terpenes, including eucalyptol and α-pinene, display significant antimicrobial properties against various bacteria [39]. Studies also explore the potential of terpenoids in inhibiting the growth of Gram-positive bacteria associated with endocarditis, although further research is needed for clinical application [40]. Periodontal disease, prevalent in companion animals, is currently treated with chlorhexidine, which has reported side effects. Terpenes, such as β-caryophyllene and linalool, show promise in inhibiting bacterial growth, biofilm formation, and enzymatic activities crucial for plaque formation and caries development [41]. Terpenoids from propolis, like apigenin and farnesol, hinder biofilm formation and reduce dental caries incidence in rats infected with *Staphylococcus sobrinu*s. Other diterpene derivatives, such as ent-karate and ent-pimarane, also exhibited the ability to inhibit the growth of dental caries pathogens [42].

The exploration of natural product alternatives to antibiotics has gained significant momentum in the agricultural sector, where the routine use of antibiotics in animal feed is being reconsidered, leading to an increased focus on alternatives such as terpenoids. Terpenes like cinnamaldehyde, carvacrol, eugenol, and thymol have comparable effects to antibiotics in promoting animal growth. Studies have illustrated their ability to enhance daily weight gain in swine and poultry, rivalling the effects of traditional antibiotics. While the modulation of gut microbial ecology and the reduction of pathogenic strains through essential oils have shown promise, the outcomes of trials incorporating these oils into animal diets have been varied. In the poultry industry, eucalyptus extracts have positively impacted production and immune stimulation in laying hens [43]. This surge in interest aligns with organic farming standards restricting synthetic antibiotic use, leading to a growing inclination towards plant-based antimicrobials for organic practices. Notably, terpenes derived from plants like thyme and oregano have demonstrated potent activity against various Gram-positive bacteria affecting intensive poultry farming, showing promise in combating avian Streptococcosis and presenting a potential solution as feed additives to counteract these infections in birds. Terpenes, such as thymol, eugenol, and curcumin, have demonstrated antimicrobial activity against Gram-positive bacteria such as *Clostridium perfringens* prevalent in animals’ GI tracts. Studies indicate the efficacy of terpene blends in controlling *C. perfringens* in broiler chickens’ intestines, suggesting the potential to prevent GI infections and necrotic enteritis [44]. The anti-inflammatory properties of terpenes further enhance their appeal for managing GI infections. In ruminants, where the gut microbiota plays a crucial role in GI health, terpenoids like eugenol and capsaicin show promise in controlling microbial populations and reducing inefficiencies in rumen fermentation. Bovine mastitis, another prevalent and economically burdensome condition in the agriculture sector, often involves Gram-positive bacteria like *S. aureus* and *Streptococcus* species. Terpenes offer a compelling alternative to traditional disinfectants, with citral, geraniol, and limonene demonstrating promising effects against mastitis-causing pathogens. Aiemsaard demonstrated the inhibitory effects of citral and geraniol on biofilm formation and structural organisation of *S. aureus* cells [45]. Additionally, Cerioli highlighted the inhibitory effects of limonene and its potential application in vaccine therapy against bovine mastitis.

In the fishery and aquaculture industry, terpenes, including linalool and nerolidol, have demonstrated potent antibacterial activity against common aquatic pathogens [46]. These terpenes also exhibit immunostimulatory effects, enhancing the immune response of aquatic species and reducing susceptibility to infections. Their low toxicity and biodegradability make them environmentally friendly alternatives to synthetic antibiotics, aligning with sustainable aquaculture practices. As demonstrated by [47], nanotechnology presents a promising avenue to enhance the effectiveness of natural bioactive compounds like nerolidol, showcasing reduced mortality rates and increased survival in infected fish.

While terpenes may not entirely replace antibiotics in veterinary medicine, their multifaceted properties position them as valuable adjunct therapies or alternatives. Understanding their precise interaction mechanisms with Gram-positive bacterial structures and defences is crucial for unlocking their full therapeutic potential. It offers a transformative approach to bacterial infection treatments in veterinary practices.

### Mycobacteria

The use of terpenes as a potential treatment for mycobacterial infections has garnered significant attention due to the rise of AMR among *Mycobacterium* species. *Mycobacterium* is a genus of bacteria within the family *Mycobacteriaceae*, characterised by their unique cell wall structure and slow growth rate [48]. *Mycobacterium* encompasses various species, causing diseases such as bovine tuberculosis, avian tuberculosis, and paratuberculosis (Johne’s disease), leading to economic losses and health risks for animals and humans [48]. Terpenes have displayed antimycobacterial properties against various *Mycobacterium* strains, as demonstrated by studies investigating their effects on bacterial activity and drug resistance. Compounds like limonene, carvone, thymol, and carvacrol have shown promising antimycobacterial activity, displaying low MICs against *Mycobacterium tuberculosis* and other strains in vitro. Additionally, studies have highlighted the synergistic effects of terpenes with conventional antimycobacterial drugs, potentially enhancing their efficacy against *Mycobacterium* species [49]. Although the findings show promise, further research is essential to understand the mechanisms of action, optimise formulations, and evaluate the safety and efficacy of terpenes as a viable treatment option for mycobacterial infections in veterinary settings.

### Gram-Negative

Gram-negative bacteria are also a concern to the veterinary industry, affecting livestock, pets, and wildlife, causing various diseases, and posing challenges in treatment and management. Several Gram-negative bacteria are highly prevalent in veterinary medicine, including *Escherichia coli*, *Salmonella spp., Pasteurella multocida,* and *Pseudomonas aeruginosa* [50]. Many of these bacteria are the causative agents of a variety of diseases in animals, such as urinary tract infections, respiratory infections, septicaemia, enteritis, mastitis, and wound infections [50]. However, the availability of antimicrobial treatments, free from resistance against Gram-negative bacteria in veterinary settings, is becoming increasingly limited [3].

Terpenoids’ ability to disrupt membrane integrity is particularly pronounced in Gram-negative bacteria, which possess lipopolysaccharides in their cell walls, creating a hydrophilic surface. Lipophilic terpenes have been shown to cause the disintegration of the outer membrane and disrupt the cytoplasmic membrane of Gram-negative bacteria, overcoming the resistance posed by their cell walls. Oxygenated terpenes, in particular, have shown potent antibacterial effects, surpassing the effectiveness of traditional antibiotics [12]. Scanning electron microscopy (SEM) images indicate that terpenes induce cell death by compromising cellular membrane integrity or function [12].

**Fig. 4:**
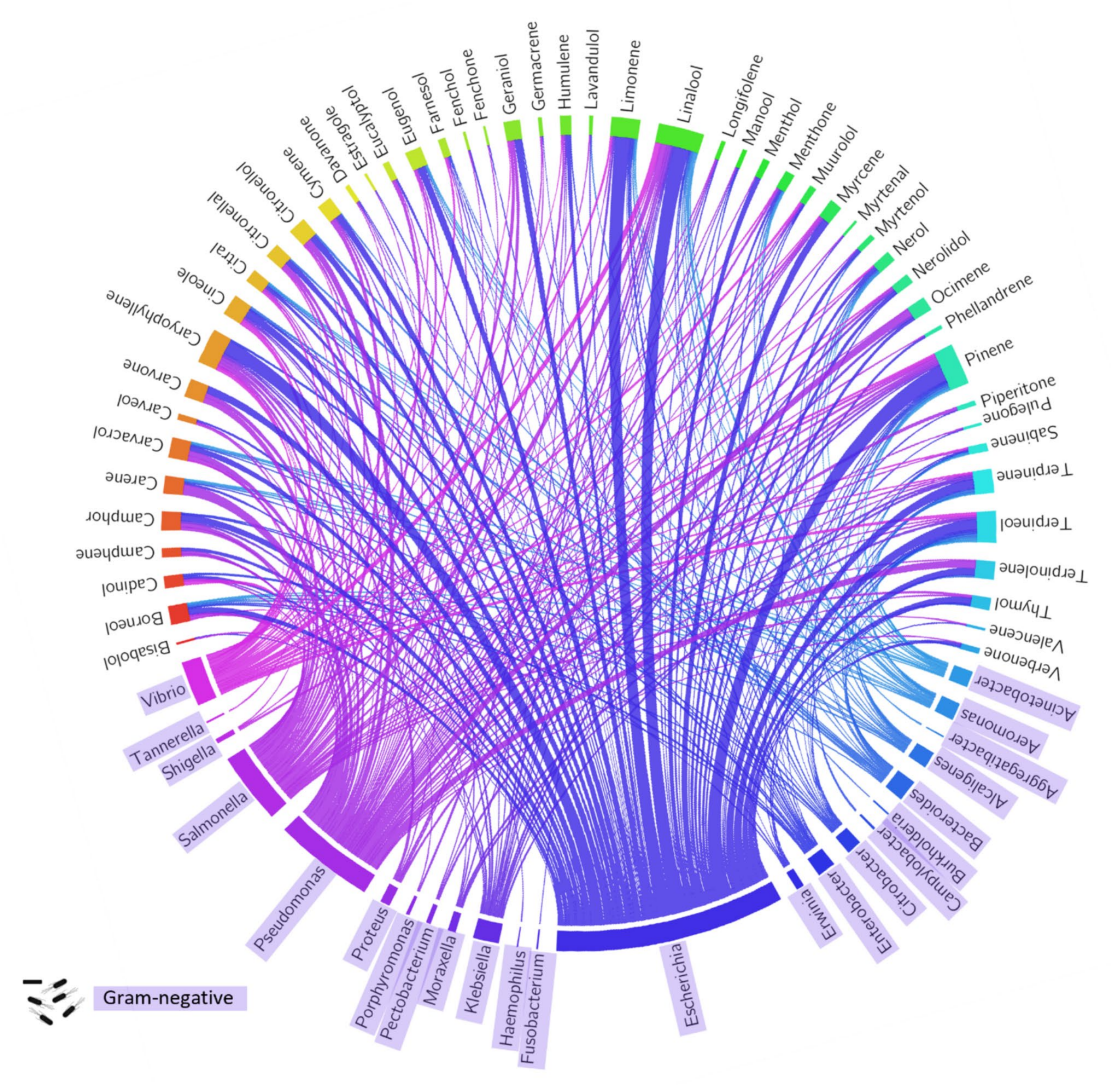
Interaction between the diversity of terpenoids and Gram-negative bacteria based on a survey of peer-reviewed articles on antimicrobial terpenes. The circus plot illustrates the relationship between various terpenoids tested for their antibacterial activity against specific Gram-negative bacteria, as reported in the literature. The data were compiled from a systematic survey of studies examining the antimicrobial activity of terpenoids, and the connections in the plot highlight the terpenes tested against each bacterial genus. The plot was generated using the Circos visualisation software, with the input dataset derived from the data available in the supplementary materials

In various studies, terpenoids have demonstrated efficacy against common veterinary microbial pathogens (Fig. 4), with carvacrol standing out as a potent antimicrobial agent. Terpenoids extend their antimicrobial prowess to combat GI infections caused by Gram-negative bacteria, urinary tract infections in pets, and respiratory infections in poultry. These natural compounds also show potential in addressing veterinary dermatology and periodontal infections, offering solutions for reduced inflammation, pruritus, and dental plaque control in canine species. The agricultural perspective also highlights the potential of terpenoids in combating Gram-negative bacterial infections in poultry and also exhibit effectiveness in managing mastitis in dairy cattle, inhibiting *Escherichia coli* biofilm formation and mature biofilms caused by mastitis pathogens, which can be a significant contributor to the pathogenicity of the infection [51]. The effectiveness of terpenoids extends to biofilm disruption, as demonstrated in the case of farnesol against *Burkholderia pseudomallei* biofilms [52].

Delving deeper into individual terpenes reveals their specific antimicrobial activities. Linalool, a monoterpene alcohol, consistently demonstrates broad-spectrum antimicrobial properties, exhibiting synergistic interactions with antibiotics and potential reversal of antibiotic resistance. Limonene, another cyclic monoterpene, showcases antibacterial, anti-inflammatory, and anti-tumour activities, making it a potential natural preservative in food. Carvacrol and thymol exhibit strong activity, particularly against Gram-negative strains. It has been indicated that these lipophilic terpenes can cause the disintegration of the outer membrane and disrupt the cytoplasmic membrane of Gram-negative bacteria. Studies on pinene, nerolidol, caryophyllene, and geraniol highlight their antimicrobial potential against Gram-negative bacteria, offering unique mechanisms of action. Caryophyllene mainly shows selective antibacterial activity, apoptosis induction, and potential use in dogs’ combating bacterial dental plaque formation. Overall, there is significantly limited in vivo data supporting the possible use of terpenoids as an antibiotic alternative (Table 1), highlighting where the future of this work should progress.

**Table 1:** In vivo testing of terpenes

Terpene	Infection model	Microbes examined	Experimental method	Terpene test conc	Efficacy	References
Nerolidol	Nile tilapia juveniles	*S. agalactiae*	Relative percent survival Brain tissue bacterial load count Brain Oxidative stress	1.5 mg/kg	Reduced brain bacterial load levels (*F* 1,70 = 4.45)	[53]
Farnesol	Humans	*S. aureus*	Clinical response Biophysical assessment of skin surface Skin microflora counts	0.02%	Reduced by 1.2 ± 1.74 log(cfu/cm^2^)	[36]
Thymol, Eugenol, Curcumin, Piperin	Broiler chickens	*C. perfringens*	Intestinal and faecal bacterial counts	100 ppm	Reduced by 22.5% for faeces, 23.4% for jejunum, 40.1% for cloaca and 11.8% for caecum	[44]
Thymol, Carvacrol, Eugenol, Curcumin, Piperin				100 ppm	Reduced by 17.5% for faeces, 24.4% for jejunum, 24.4% for cloaca and 9.9% for caecum	
β-Caryophyllene	Mongrel dogs	*Enterococcus spp.* *Streptococcus spp.* *Aerococcus spp.* *Bacillus spp.* *Lactococcus spp.*	Relative percent teeth plaque cover	50 mg/ml	Reduced by 23.3 ± 2.6%	[54]
Linalool	Neutropenic rat thigh	*E. coli*	Bacterial colony counts	100 mg/kg	Reduced by 4.07 ± 0.05 Log^10^cfu/ml	[55]

## Terpenes as Antiparasitic Agents

**Fig. 5:**
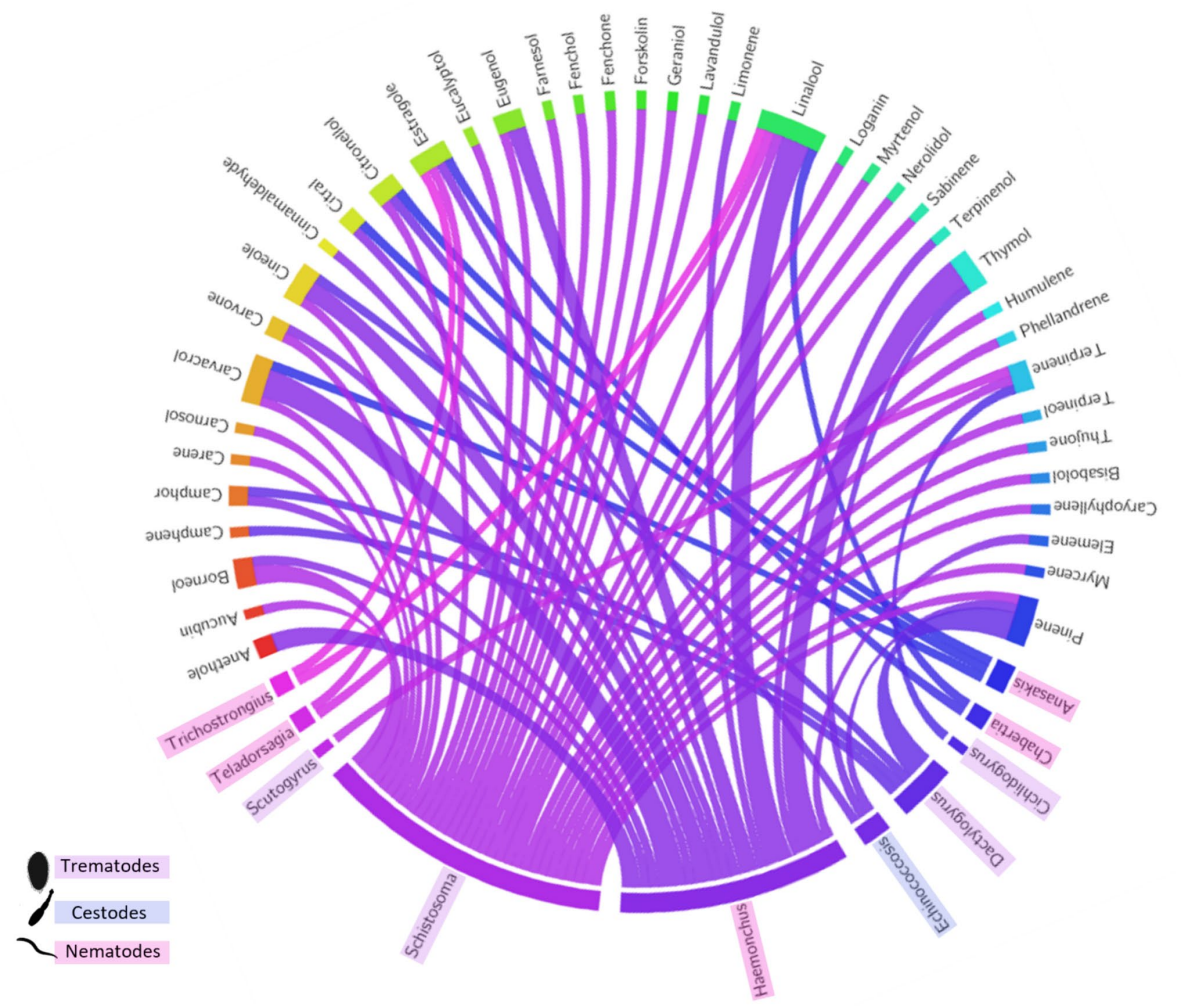
Interaction between the diversity of terpenoids tested on helminth parasites based on a survey of peer-reviewed articles on antiparasitic terpenes. The Circos plot illustrates the relationships between various terpenoids and specific helminth parasites, as reported in the literature. The data were compiled from a systematic survey of studies examining the anthelmintic activity of terpenoids, and the connections in the plot highlight the terpenes tested against particular helminth genus. The plot was generated using the Circos visualisation software, with the input dataset derived from data available in the supplementary materials

The prolonged use of chemical anthelmintics has led to resistance and environmental concerns, prompting interest in plant extracts, including terpenes, as alternative sources for parasite treatments [56]. Terpenes offer significant potential as antiparasitic alternatives in veterinary medicine, targeting many parasites, from protozoa to metazoans (Fig. 5). One notable advantage of terpenes is their resistance profile, as screens for terpene-resistant variants have yet to uncover significant resistance in target pathogens. Additionally, terpenes have broad membrane-disrupting capabilities, leading to chemo-osmotic stress and generalised death of their targets.

### Helminths

Infections due to helminth parasites are common in the veterinary industry, affecting domestic animals, wildlife and livestock, resulting in severe economic losses. Helminth infections comprise members from the phyla trematoda (flukes), cestoda (tapeworms), and nematoda (roundworms). Infections with parasitic helminths represent a significant economic and welfare burden to the global livestock industry. The current strategy of the World Health Organisation to prevent helminth infection includes increasing hygienic awareness, providing better sanitation and preventative anthelmintic drug therapy in vulnerable populations. Nowadays, anthelmintic drugs are used heavily in livestock, both in case of infection and as a preventative measure [57]. However, this has led to the development of resistance against several of the most common drugs, such as levamisole, ivermectin, and thiabendazole. As many as 70% of the livestock in developed countries now has helminths that are drug resistant, and multi-drug resistance is common [58]. Because of this, novel anthelmintics are urgently needed to help combat large-scale production losses. Terpenes have garnered interest as potential novel treatment options due to their diverse biological activities, including antiparasitic properties. While the literature suggests that terpenes have anthelmintic properties against various veterinary helminth infections, it is essential to note that their efficacy can vary depending on factors like parasite species, dosage, and formulation (Table 2). Terpenes are often considered complementary or alternative treatments to synthetic anthelmintics, particularly in cases where drug resistance is a concern.

**Table 2:** In vivo efficacy of terpenes against helminth infections

Terpene	Infection model	Microbes examined	Experimental method	Efficacy	References
Carvacrol	Mice, Sheep	*H. contortus*	EHT LDT AWMT Toxicity (mice) FECRT (sheep)	LD10 = 546.8 mg/kg LD50 = 919 mg/kg	[64]
Thymol	Mice	*H. contortus*	EHT LDT	Ovicidal EC50 = 0.69 mg/ml Larvicidal EC50 = 2.11 mg/ml	[70]
Anetol	Sheep (Experimentally infected)	*H. contortus*	EHT LDT	Ovicidal EC50 = 0.55 mg/ml Larvicidal EC50 = 2.49 mg/ml	[70]
Thymol	Goats (Experimentally infected)	*H. contortus*	EHT LMT LDT AWMT FECRT (sheep)	Testing conc. = 5 mg/ml EHT = 96.4–100% LDT = 90.8–100% LMT = 97–100% AWMT = 100% (< 8 h)	[66]
Linalool	Sheep (Naturally infected)	GIN	EHT	MIC = 0.5% (v/v)	[71]
Estragole		GIN	EHT	MIC = 3.125 mg/ml	[67]

*GIN* Gastro-intestinal nematodes, *EHT* Egg hatch test, *LDT* Larval development test, *LMT* Larval motility test, *AWMT* Adult worm motility test, *FECRT* Faecal egg count reduction test

#### Trematodes

The potential use of terpenes in treating trematode infections in the veterinary industry has garnered attention due to their promising antiparasitic properties. Trematodes, or flukes, are parasitic flatworms that infect various animals, including livestock and companion animals. Terpenes, derived from plants, have shown potential as antiparasitic agents against various trematode species. Several studies have explored the potential of terpenes as treatments for trematode infections in the veterinary industry. Research on how terpenoids inhibit trematodes is still in its infancy; however, preliminary research showcasing the efficacy of plant extracts such as *Rosmarinus officinalis (*Rosemary), *Cymbopogon citratus* (Lemongrass), *Conyza canadensis* (Horseweed), and a variety of Zimbabwean plant extracts, high in terpenoids to treat various trematode species including *Schistosoma mansoni*, *Fasciola gigantica, Paramphistomum cervi*, *Hymenolepis diminuta,* and *Dactylogyrus minutus*, hold promise for the likelihood of terpene being the active component responsible for their anthelmintic activity [59]. In fact, Zoral et al. [60] highlighted 1,8-cineole as responsible for rosemary extract effects against the monogenean parasite—*Dactylogyrus minutus*, both in vitro and in vivo. More in-depth studies into the anthelmintic activity of individual terpenes yielded variable results in vitro. In vivo work by Morales-Serna et al. [61] examined α-terpinene and ( +)-limonene oxide against ancyrocephalid monogenean parasites in *Nile tilapia* fish, noting ( +)-limonene oxide’s high efficacy, demonstrating 90% mortality at 55.4 mg/L in a 5-h treatment. Other rarer terpenoids have also been examined, such as the diterpenoid 7-keto-sempervirol, which revealed dual anthelmintic activity against *Schistosoma mansoni* and *Fasciola hepatica* [62]*,* showcasing potential as a starting point for anthelmintic drug development with specific effects on both larval and adult trematodes. Regarding the anthelmintic mechanism of action, Shafiq et al. [63] proposed that terpenes are involved in tegumental membrane disintegration due to their lipophilic compounds, which may result in disturbing normal bodily biochemical and physiological processes. This work collectively showcases the potential of various terpenes in combating trematode infections, emphasising their diverse effects and potential as alternative treatments in veterinary medicine.

#### Nematodes

The potential use of terpenes and their derivatives for treating nematode infections in the veterinary industry represents an innovative and promising avenue for combating resistant parasitic infestations. Numerous studies have demonstrated the efficacy of certain terpenes in exhibiting significant anthelmintic activity, disrupting the life cycle of nematodes and reducing parasite burden. Carvacryl acetate (CA), derived from carvacrol, demonstrated significant anthelmintic efficacy in both in vitro and in vivo assays [64]. The acetylated product CA exhibited substantial inhibition of larval hatching, larval development, and adult worm motility against *Haemonchus contortus*. Moreover, CA showed lower acute toxicity than its precursor, carvacrol, emphasising its potential safety profile. On the other hand, *Cymbopogon schoenanthus* essential oil, composed mainly of geraniol, failed to demonstrate significant anthelmintic effects in an experimental study with lambs infected with multi-drug-resistant *H. contortus* [65]. Despite the absence of statistically significant reductions in faecal egg count and total worm burden, the study highlighted the safety of the essential oil in sheep. Several other studies have focussed on terpenes from different plant sources [66]. These compounds exhibited various degrees of ovicidal and larvicidal activity against *H. contortus*. Thymol showed good efficacy against different stages of *H. contortus*, validating the use of phenolic terpenoids as an anthelmintic agent [66]. In contrast, the study by Katiki et al. [65] emphasises the importance of carefully considering experimental design and dosage. This study’s lack of significant anthelmintic effects may be attributed to factors such as dosage regimen, experimental conditions, or the specific resistance profile of the *H. contortus* strain used. While in vitro studies, such as that by Štrbac et al. [67] evaluating a mixture of linalool and estragole against sheep gastrointestinal nematodes (GIN), show promise, the translation to in vivo efficacy needs thorough investigation. In vitro results may not fully capture the complexity of host–parasite interactions and the challenges presented in a living organism. Additionally, the potential development of resistance over time should be considered when evaluating the long-term efficacy of terpenes.

It appears that the mechanisms through which terpenes exert their anthelmintic effects involve interference with crucial physiological processes in nematodes. Terpenes disrupt neurotransmission, interfere with energy metabolism, and damage nematodes’ cuticle, impeding their ability to survive and reproduce. This multifaceted approach makes terpenes a promising class of compounds for nematode control. The functional groups of terpenes play a pivotal role in determining their anthelmintic activity. For instance, the hydrophobic nature of certain terpenes facilitates the penetration of the nematode cuticle, enhancing its efficacy. Oxygen-containing functional groups, such as those found in cineole, disrupt biochemical pathways critical for nematode survival. Terpenes diverse modes of action offer a potential solution to the issue of resistance to current anthelmintic treatments. Combining terpenes with other anthelmintic agents or rotating their use with different terpenoids may help mitigate resistance development. Additionally, understanding the genetic basis of resistance in nematodes can guide the rational design of terpene-based therapies.

#### Cestodes

Research on the potential use of terpenes to treat cestode infections, particularly hydatidosis or cystic echinococcosis caused by *Echinococcus granulosus*, while still preliminary, has shown promising results. The existing treatment strategies using benzimidazoles suffer from limited efficacy and variable responses, necessitating the exploration of new drugs. Essential oils and their components, especially terpenes like β-pinene, citronellol and thymol, have exhibited scolicidal effects against *E. granulosus *in vitro [68]. Thymol, at a concentration of 5 µg/ml, demonstrated significant inhibitory effects on cell viability. This viability reduction was coupled with cell number alterations, cell collapse, and disruption of the normal tridimensional composition of cellular aggregates [69]. β-pinene and citronellol also exhibited high scolicidal activity, suggesting their potential as candidate ingredients for developing green scolicidals [68]. Once again, it is suggested that the diverse and multifaceted nature of terpene action could potentially mitigate resistance development. By targeting different constituents or using a combination of terpenes, there may be a decreased likelihood of cestodes developing resistance compared to traditional treatments, offering promising avenues for pharmaceutical alternatives (Table 2).

### Ectoparasites

The rising concern surrounding ectoparasites in companion animals, livestock, and wildlife stems from their significant impact on health, productivity, and zoonotic disease transmission. These parasites, like fleas, ticks, lice and mites, not only cause direct harm and discomfort but also act as vectors for diseases, affecting animals’ well-being and, in some cases, entire ecosystems. The potential use of terpenes as treatments for these infections has gained attention due to their natural origin, efficacy, and lower risk of resistance compared to traditional chemical agents. The sale and use of ectoparasiticides to control arthropod parasites in domestic animals constitute a significant sector of the global animal health market. Animals are infected by several parasitic insect and acarine species, causing considerable economic losses in livestock production, intense irritation and skin disease in companion animals, or public health issues, including bites of humans or zoonotic disease transmission. Dog and cat fleas, for example, can be a severe source of both animal and human atopic dermatitis (eczema), which has led to a rapid expansion in the development of flea control products. The control of ectoparasite infections of veterinary importance still relies heavily on using chemicals that target the arthropod nervous system. Such compounds have suffered from several drawbacks, including the development of resistance and concerns over human and environmental safety. However, the search for safer technologies has been hindered by the limited number of active target sites present in arthropods and, to some degree, by the ever-increasing costs of research and development of compounds with novel modes of action.

#### Ticks

**Fig. 6:**
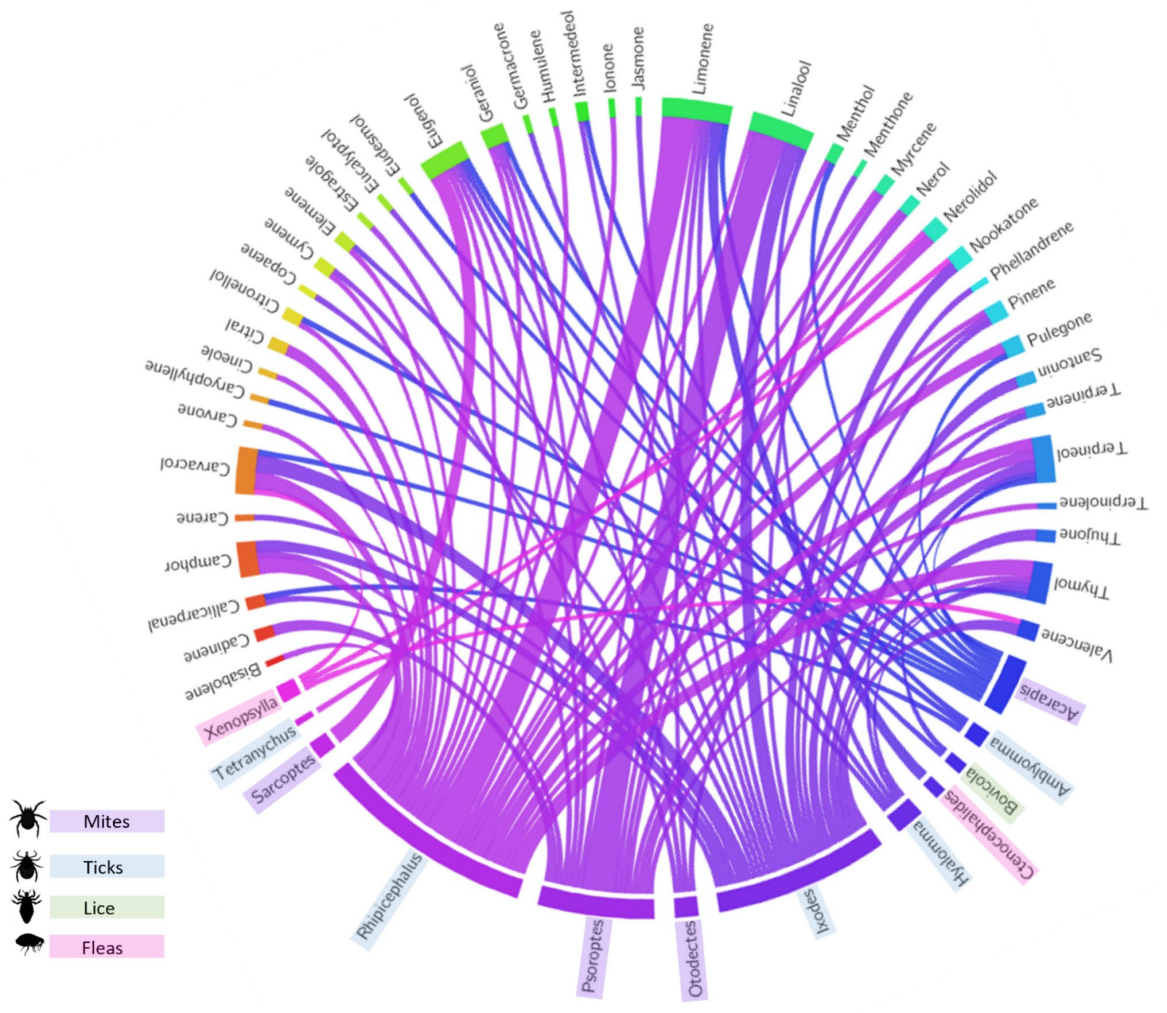
Interaction between the diversity of terpenoids and ectoparasites based on a survey of peer-reviewed articles on antiparasitic terpenes. The Circos plot illustrates the relationships between various terpenoids and specific ectoparasites, as reported in the literature. The data were compiled from a systematic survey of studies examining the antiparasitic activity of terpenoids, and the connections in the plot highlight the terpenes tested against the parasite genus. The plot was generated using the Circos visualisation software, with the input dataset derived from data available in the supplementary materials

The use of terpenes and their derivatives, specifically terpenoids, in treating tick infections within the veterinary industry holds great potential. Ticks are notorious ectoparasites that pose significant threats to animal health, transmitting various pathogens and causing diseases in companion and livestock animals [72]. With resistance to conventional acaricides on the rise, alternative treatments, including terpenoids, are being explored due to their promising insecticidal and repellent properties. Numerous studies have identified terpenes with efficacy against ticks, showcasing their acaricidal and repellent effects (Fig. 6). The terpenoids, callicarpenal, and intermedeol showed high repellent activity against Ixodes scapularis and *Amblyomma americanum* nymphs [73]. Camphor demonstrated a synergistic acaricidal effect on *Rhipicephalus sanguineus*, resulting in significant mortality of adult ticks and larvae [74]. Carvacrol has shown significant acaricidal efficacy. For instance, in the case of *Rhipicephalus turanicus*, carvacrol killed all ticks within 6 h of exposure [75]. Additionally, thymol exhibited a deleterious effect on *Rhipicephalus sanguineus* larvae, especially on engorged larvae, resulting in 100% mortality at higher concentrations [76]. Moreover, nerolidol and limonene displayed notable acaricidal activity against *Rhipicephalus microplus* larvae and engorged females [77]. Interestingly, (-)−10-epi-γ-eudesmol demonstrated strong repellent activity against the lone star tick, *Amblyomma americanum*, showcasing the potential of natural terpenoids as tick repellents [78]. The findings suggest that terpenes such as carvacrol, thymol, callicarpenal, and (-)−10-epi-γ-eudesmol hold promise in tick control. However, while these studies collectively highlight the efficacy of various terpenes against ticks, it is essential to note that the effectiveness can vary based on the tick species and the specific terpene or terpenoid. The mechanisms of action often involve disruption of tick development, inhibition of egg-laying, and direct mortality across different life stages.

#### Fleas

Fleas (*Siphonaptera*) are widespread ectoparasites posing significant concerns in veterinary medicine due to their prevalence and potential as disease vectors. *Ctenocephalides felis* [79] is the most prevalent species in companion animals. Many fleas are vectors of diseases like Rickettsiosis and Bartonellosis, raising concerns about the spread of disease among animals and humans [79]. Flea-allergy dermatitis and disease transmission exacerbate the problem, necessitating effective flea control methods. Chemical insecticides, repellents, and endectocides constitute a significant portion of the veterinary pharmaceutical market. However, concerns about antiparasitic resistance and environmental impact have fuelled interest in natural alternatives like plant terpenoids. Studies have highlighted the potential of specific terpenoids—limonene, eugenol, carvacrol, and nootkatone—found in essential oils like citrus, clove, *Schinus molle*, and Alaska yellow cedar, showing promise against fleas [80]. These terpenoids have demonstrated varying efficacy against flea stages, exhibiting insecticidal activity, development inhibition, and residual effects (Fig. 6). Moreover, botanical formulations like Bioticks®, containing thyme, rosemary, lemon balm, fenugreek, wormwood, and lemongrass extracts, have shown potential in reducing flea populations in pets [81]. However, challenges remain regarding standardisation, residual efficacy, and long-term suitability, necessitating further research into natural compounds for sustainable flea control strategies in veterinary medicine [81].

#### Mites

Plant-derived terpenoids have shown substantial promise in combatting various parasitic mites (Fig. 6). Mange, caused by *Sarcoptes or Psoroptes spp.*, presents a significant challenge in many wild and domestic mammal species [82]. Studies have highlighted the acaricidal activities of several terpenes against sarcoptic mange mites. Research conducted by Pasay et al. [82] demonstrated the efficacy of eugenol-based compounds against *Sarcoptes scabiei* mites, exhibiting toxicity levels comparable to positive control acaricides, which present their own drawbacks with resistance increasing. Li et al. [83] also found six terpenes, including carvacrol, eugenol, and geraniol, to possess significant ovicidal activities against *S. scabiei* eggs. Furthermore, investigations involving *Psoroptes spp.* revealed the acaricidal potential of various terpenoids. Studies by Perrucci et al. [84] indicated that terpenes possessing free alcoholic or phenolic groups displayed potent acaricidal activities against *Psoroptes cuniculi;* in fact, thymol and eugenol killed nearly 100% of parasites at all dosages used in the direct contact tests, indicating that the phenol functionality enhances the miticidal activity of terpenes. Linalool was also shown to display acaricidal properties both in vitro and in vivo [84]. Guo et al. [85] and Wall and Bates [86] also investigated the acaricidal effects of terpenoids, including cinnamic acid and δ-cadinene against *Psoroptes ovi*s and P. *cuniculi*, respectively, showcasing promising outcomes. Moreover, research has turned to terpenoids as a much-needed alternative treatment for *Dermanyssus gallinae*, the poultry red mite, a haematophagous ectoparasite of poultry implicated as a vector of several major pathogenic diseases. Various studies have indicated the potential of terpenoids like eugenol, geraniol, and citral as effective [87]. These findings suggest that plant terpenoids possess substantial acaricidal properties and may serve as potential alternatives in managing various mite infections in animals, addressing issues related to resistance and offering promising therapeutic approaches.

One of the great successes of commercialising terpenes as an antiparasitic agent is the use of thymol formulations to treat Varroa mites. Varroa mites (*Varroa destructor* and *V. jacobson*i) are an ectoparasite of the honeybee, *Apis mellifera*, considered to be the most invasive pest of honeybees worldwide and a significant threat to the apiculture industry. Initially, studies on monoterpenoids, with a focus on thymol, emerged as a promising treatment for controlling mite infestations. Ellis [88] explored various monoterpenoids and found thymol numerically more toxic to mites. This work was followed up by Imdorf et al. [89] who reestablished the efficacy of thymol, demonstrating high mortality with low residual build-up in honey, this work went on to develop ‘Apilife VAR©’ and ‘Apiguard©, varroacide products with thymol as the active ingredient. Apilife and other thymol-based blends offered an alternative to the current treatment method of strips containing the pyrethroids, which have resulted in residues accumulating in the wax, jeopardising the quality of bee products. Thymol formulations like Apiguard have been studied extensively and praised for their efficacy, simplicity of application, and compatibility with their active components with food products. While some concerns have been raised regarding the effects of thymol products on colony development, bee behaviour, and potentially genotoxic effects, combining biotechnical methods of thymol application can provide safe and effective therapy against Varroa spp. [90]. The diverse formulations and applications of thymol underscore its potential as a tool for integrated pest management in combating Varroa mites while also urging continued research to optimise its use and address potential drawbacks.

#### Lice

Lice infestations in animals’ hair, skin, scales, and feathers can occur in any environment; however, they are particularly prominent in production animals such as cattle when crowded together in winter [91]. Due to the cost associated with lice treatments, farmers are interested in assessing new chemicals to control lice of veterinary importance [91]. Preliminary research has begun examining plant-derived terpenoids in both in vitro and in vivo studies (Fig. 6), primarily concentrating on chewing lice within the *Bovicola* genus. Certain terpenes are highly effective against both lice and lice eggs; a patent from 2007 [92] highlighted the use of the terpene aldehyde citral, extracted from lemongrass essential oil, and its combination with other terpenes are highly effective in killing lice and their eggs, as well as ants, mites, and other parasites. It was proposed that the mode of action of these aqueous terpene formulations was by direct solvent action on the wax-containing epicuticle of the chitinous exoskeleton of lice. Studies focussing on chewing lice (*Bovicola ovis* and *B. ocellatus*) showcased the remarkable efficacy of terpinen-4-ol, the major component of tea tree oil (*Melaleuca alternifolia*) [93]. Tests demonstrated high mortality rates of lice and eggs through immersion, vapour exposure, and wool application. Moreover, field studies with sheep exhibited significant reduction or elimination of lice following treatment with tea tree oil formulations. Similarly, other essential oils, lavender (*Lavandula angustifolia*), peppermint (*Mentha piperita*), eucalyptus (*Eucalyptus globulus Labillardiere*), clove bud (*Eugenia caryophyllata*), and camphor (*Cinnamomum camphora*), displayed potent toxicity against lice infestations in donkeys and water buffaloes [94]. This activity was primarily attributed to the major terpenoids in the oils, showcasing the potential for developing environmentally safe pediculicides and repellents for managing ectoparasitic lice in large animals. The benefit of terpene formulations is that their observed modes of action suggest a reduced likelihood of resistance development compared to conventional pesticides [92]. In addition, terpene-based treatments are non-neurotoxic, distinguishing them from other pediculosis medications [92].

### Protozoa

The veterinary industry confronts substantial challenges in managing protozoal infections, including those caused by parasites like coccidia, trypanosomes, and others, which greatly impact animal health and productivity [95]. Although conventional treatments exist, increasing attention is being directed towards terpenes as potential novel treatment options due to their varied biological activities. Whether derived from essential oils, plant extracts, or isolated compounds, terpenoids exhibit promising attributes in disrupting protozoal life cycles, lessening infection severity, and offering alternatives to conventional treatments [95]. Numerous studies highlight the efficacy of terpenes against various protozoa, including *Giardia, Leishmania, Eimeria, Trypanosoma, Babesia, Toxoplasma*, and *Trichomonas* [95]. Terpenes, such as thymol, carvacrol, nerolidol, and α-pinene, demonstrate significant antiparasitic activity against protozoal infections. In poultry farming, terpenes from essential oils like oregano, turmeric, thyme, and sage show promise in controlling coccidiosis, a significant concern in poultry, by disrupting the life cycle of *Eimeria spp*. parasites and enhancing gut health in birds [96]. The diverse range of terpenes and their proven antiprotozoal activities offer promising avenues for future development in veterinary medicine, potentially providing safer and more effective alternatives for managing animal protozoal infections.

## Future Directions in the Development of Terpenes as New Antimicrobial Agents

The escalating interest in complementary and alternative medicine within the veterinary sector reflects a growing trend among pet owners and farmers who seek natural therapies for their beloved companions and livestock [97]. Despite the consistent demonstration of excellent bioactivities by plant-derived terpenes, several challenges must be addressed before terpenes can be successfully translated into clinical treatments. Issues such as low solubility, poor bioavailability, and potential toxicity must be addressed through formulation optimisation and safety evaluations. Additionally, the complexity of interactions between terpenes and different pathogens necessitates further research to unravel their precise modes of action and optimise their therapeutic potential. The effectiveness of terpenes is intricately tied to functional groups, with their antimicrobial actions lacking a consolidated classification in the literature. The broad spectrum of microorganisms affected raises issues regarding therapeutic potential and concerns about native host microflora imbalances. Additional challenges stem from the laborious extraction process, high costs, and variations in terpene content among plant batches. Moreover, the need for more standardisation in experimental designs and methods for assessing antimicrobial activity complicates comparisons across studies.

Microencapsulation and nanoparticle technologies are emerging as promising avenues to overcome many challenges, enhancing terpenes’ application in veterinary medicine. A study by Zahi et al.[98] highlighted the antimicrobial potential of microencapsulated carvacrol, thymol, and d-limonene, offering controlled release and broad-spectrum inhibition. Lipid-based nanocarriers, such as liposomes and solid lipid nanoparticles, have also improved terpene stability and bioavailability. Recent investigations by Darvish et al. [99] showcase the integration of limonene into low-density polyethylene films, providing sustained release and antibacterial activity. Encapsulation using yeast particles (YPs) creates water-soluble terpene materials without surfactants, allowing for sustained release and reduced dosage. Han et al. [38] utilised poly(DL-lactide-co-glycolide) (PLGA) nanoparticles for terpene encapsulation, enhancing solubility, permeation, and release. The encapsulation implementation effectively addresses the challenges associated with terpenes, offering protection from volatilisation, controlled release, and enhanced stability. Nanoencapsulation contributes to improved bioavailability, decreased toxicity, and targeted delivery, providing a potential solution to the hurdles of terpene application in veterinary medicine. Biomolecules extracted from their natural matrices exhibit instability when exposed to temperature, pH, light, and oxygen concentration changes, necessitating preserving their desired biological effects. The literature has highlighted (nano)encapsulation as a prospective method for overcoming this challenge, wherein an active substance (core material) is entrapped into a coating material. This combination allows changes in solubility and chemical and thermogravimetric stability, with several benefits, such as maintaining functionality, preventing degradation, controlling release, and ensuring food safety and shelf-life. Various encapsulation techniques are available, each dependent on factors related to the material to be protected, the matrix-forming substance, and the application conditions. Methods such as electrospinning and micelle formation are among the diverse encapsulation techniques. Nano/microencapsulation techniques have shown promise in extending the useful life of compounds obtained through plant extracts, presenting an alternative to combat degradation when exposed to various factors. For instance, the volatile terpenes in black pepper essential oil, susceptible to reduction under certain conditions, can be effectively preserved through encapsulation. Complex coacervation, with a gelatin/sodium alginate ratio of 6:1 at pH 4.0, has been identified as an ideal condition for encapsulating black pepper essential oil, showcasing good core protection and high encapsulation efficiency. Efficient encapsulation of terpenes within yeast particles (YPs) has been achieved through passive diffusion, resulting in hypercharged YP terpenes with extended payload release kinetics. This advancement holds potential applications in agriculture and pharmaceuticals, offering a delivery system with high payload capacity, increased stability, and sustained release properties. Additionally, studies exploring the encapsulation of essential oils from fruit juices, such as a terpene mixture and d-limonene, have demonstrated increased antimicrobial activity. Nanoemulsions based on food-grade ingredients prepared using high-pressure homogenisation offer effective encapsulation with varied antimicrobial capacities based on the formulation and average diameter of the delivery systems.

Another avenue to address challenges, particularly those posed by antimicrobial resistance (AMR), involves integrating terpenes with current antibiotics. Various studies have explored the synergies, additive qualities, or even antagonistic effects of this combination, offering a novel therapeutic approach to augment antibiotic efficacy, reduce dosages, and potentially overcome resistance mechanisms. Instances such as the synergy between peppermint essential oil and the antibiotic meropenem underscore the potential of these combined therapies, showcasing significant reductions in MIC values. Other terpenoids, including clerodane diterpenoid and sesquiterpenes, demonstrate notable antibiotic enhancement against pathogenic bacteria such as MRSA. Terpenes, with a molecular weight not exceeding 500 g/mol, may act as adjuvants for antimicrobials, revealing synergistic effects.

Furthermore, combination therapy holds promise in eradicating bacterial biofilm production, a significant advancement in combating persistent infections. The theories elucidating the mechanisms underlying this combinatory effect suggest a multi-targeted pharmacokinetic impact, allowing the simultaneous disruption of existing resistance mechanisms in specific pathogens. While in vitro studies yield promising results, the imperative for physiologically relevant in vivo testing remains pivotal in understanding the safety and efficacy of terpenes in conjunction with antibiotics. Unfortunately, limited data exist regarding clinical trials focussed on the antimicrobial activity of these combinations. Although the amalgamation of terpenes with existing antibiotics presents immense potential in tackling antimicrobial resistance, further research, particularly in vivo studies and clinical trials, is imperative to validate the efficacy and safety of these synergistic combinations for potential therapeutic applications.

## Conclusion and Perspectives

While the potential of terpenes as novel antimicrobial and anthelmintic treatments in veterinary medicine is evident, challenges persist. Encapsulation technologies, leveraging microencapsulation and nanoparticle strategies, emerge as practical solutions to enhance the therapeutic application of terpenes. As research progresses, these advancements promise to develop safe, effective, and controlled terpene formulations for veterinary use, aligning with the industry’s growing demand for natural and alternative treatments. Nevertheless, the exploration of terpenoids as a means to control veterinary pathogens presents an exciting avenue for future therapeutic developments. Although research in this field is still in its early stages, the substantial potential is evident. Extensive field trials, component standardisation, and extraction protocol standardisation are imperative to realise the benefits fully. The therapeutic potential of plant-derived compounds, often underestimated, requires comprehensive validation through biological tests supported by computational, in vitro, and in vivo investigations. The antimicrobial activity demonstrated by plant extracts in in vitro experiments, coupled with the renewed interest in natural therapeutics and food safety, calls for additional studies to validate their efficacy in enhancing animal performance. Despite promising results in the literature, challenges impeding the translation of terpenes into clinical treatments, such as solubility, bioavailability, and potential toxicity, demand rigorous attention through formulation optimisation and safety evaluations. The broad-spectrum activities and diverse mechanisms of action exhibited by terpenes against various stages of pathogen life cycles position them as promising candidates for innovative therapeutic strategies. However, to fully harness their potential in combating infectious diseases, further research and substantial investment are essential to overcome existing challenges and bridge the gap between current knowledge and practical clinical applications in the veterinary industry.

## Supplementary Information

Below is the link to the electronic supplementary material.

Supplementary file1 (PPTX 4802 KB)

Supplementary file2 (XLSX 116 KB)

## Data Availability

Not applicable.
