# Supplementary figures and images for: Harnessing Plant-Derived Terpenoids for Novel Approaches in Combating Bacterial and Parasite Infections in Veterinary and Agricultural Settings
Source: Curr Microbiol. 2025 Feb 12;82(4):134. doi: 10.1007/s00284-025-04113-4 (PMC11821797; doi:10.1007/s00284-025-04113-4)

## Slide 1
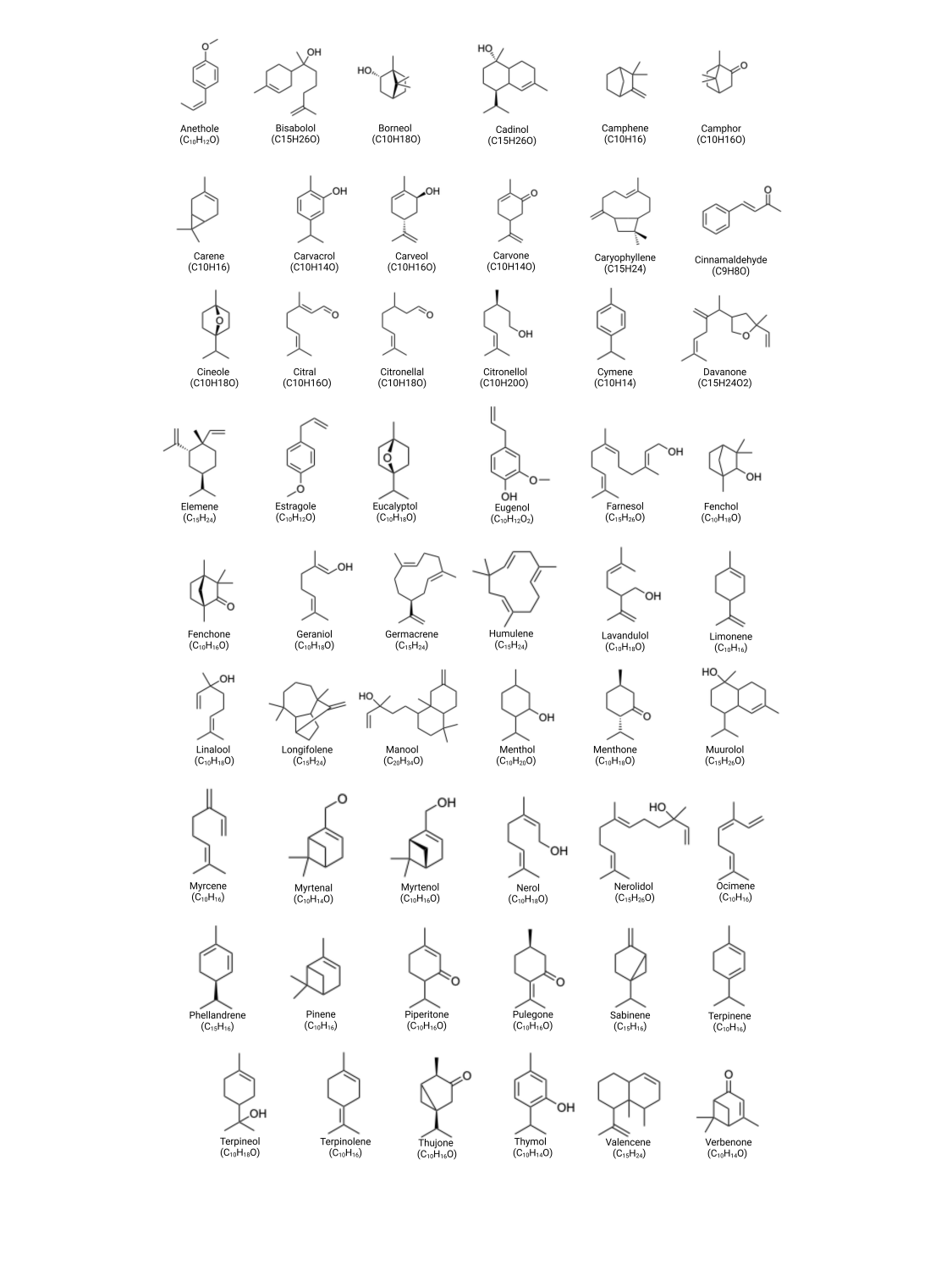

Supplement: Supplementary file 1 — Supplementary file1 (PPTX 4802 KB) [file 284_2025_4113_MOESM1_ESM.pptx]
